# *Pediococcus acidilactici* Strains Improve Constipation Symptoms and Regulate Intestinal Flora in Mice

**DOI:** 10.3389/fcimb.2021.655258

**Published:** 2021-03-18

**Authors:** Yiteng Qiao, Zhichang Qiu, Fengwei Tian, Leilei Yu, Jianxin Zhao, Hao Zhang, Qixiao Zhai, Wei Chen

**Affiliations:** ^1^State Key Laboratory of Food Science and Technology, Jiangnan University, Wuxi, China; ^2^School of Food Science and Technology, Jiangnan University, Wuxi, China; ^3^College of Food Science and Engineering, Shandong Agricultural University, Tai’an, China; ^4^National Engineering Research Center for Functional Food, Jiangnan University, Wuxi, China; ^5^Wuxi Translational Medicine Research Center, Jiangsu Translational Medicine, Research Institute Wuxi Branch, Wuxi, China

**Keywords:** *Pediococcus acidilactici*, bacteriocin, constipation, intestinal flora, regulation

## Abstract

Constipation is a prevalent gastrointestinal disorder that seriously reduces the quality of life. Clinical studies have shown that a great change or severe imbalance occurs in the intestinal microbiota of people with constipation. This study explored whether bacteriocin-producing and non-bacteriocin-producing *Pediococcus acidilactici* strains resulted in differences in the alleviation of constipation and changes in the fecal flora in BALB/c mice. The constipation-related indicators, gastrointestinal regulatory peptides and gut microbiota were identified to evaluate their alleviating effects and underlying mechanisms. The time to the first black-stool defecation and the gastrointestinal transit rate in constipated mice were found to be somewhat improved by four *P*. *acidilactici* strains (*P* > 0.05). Moreover, there were significant differences in the level of most gastrointestinal regulatory peptides in the serum, as well as in the composition and abundance of intestinal microbiota in different groups (*P* < 0.05). At the phylum level, the relative abundance of Firmicutes was significantly increased, but those of Bacteroidetes and Proteobacteria were significantly reduced after the administration of four *P*. *acidilactici* strains for 14 d (*P* < 0.05). The levels of *Bacteroides* and genera from *Enterobacteriaceae* were significantly decreased, whereas *Bifidobacterium* and *Lactobacillus* were upregulated when bacteriocin-producing *P*. *acidilactici* CCFM18 and CCFM28 strains were provided in the diet (*P* < 0.05). The results indicated that although constipation-related symptoms were alleviated to only a limited degree, the administration of four *P*. *acidilactici* strains effectively regulated the gut flora and provided a potential health benefit to the host, especially the bacteriocin-producing *P*. *acidilactici* strains.

## Introduction

Constipation, a common gastrointestinal disorder, is characterized by low defecation frequency, prolonged emptying time of the gastrointestinal tract, and incomplete defecation, which causes serious distress for patients of different ages and genders (Bharucha, 2007; Zhao and Yu, 2016). Globally, the prevalence of constipation is showing an increasing trend with changes in dietary habits, lifestyle, and psychological factors. The treatment of constipation is challenging. Irritant drugs have limited efficacy and serious adverse effects (Bharucha et al., 2017; Luthra et al., 2019). Therefore, oral supplementation with probiotics (safe and long-acting) is attracting considerable interest among gastrointestinal physicians and researchers (Dimidi et al., 2020).

The term probiotics refers to live microorganisms that are introduced to the gastrointestinal tract of the host with beneficial effects (Bezkorovainy, 2001). The oral intake of probiotics cultured *in vitro* prompts regulation of the composition and abundance of intestinal flora and the metabolism of short-chain fatty acids (SCFAs) by healthy colonic populations to inhibit the growth of pathogens, reduce the concentration of several metabolic products (such as some phenols), lower the intestinal pH, and promote intestinal peristalsis (Possemiers et al., 2009). In a double-blind placebo-controlled, randomized study of 45 children with chronic constipation, the administration of *Lactobacillus casei rhamnosus* Lcr35 resulted in increased defecation frequency, enhanced treatment success rates, decreased use of glycerin enemas, and reduced number of hard stools, compared with the placebo group (Bu et al., 2007). The beneficial effect of the consumption of probiotics has also been examined in adults and the elderly (Zaharoni et al., 2011; Eskesen et al., 2015).

*Pediococcus acidilactici* probiotic strains have been proven to produce bacteriocin from ribosomes. Since the 1980s, researchers have conducted many studies on the discovery and production of bacteriocin. Bacteriocins can colonize and persist in the human gut while having a beneficial effect on the host, including modulation of the composition of the gut microbiota, improvement of the host immune response, and enhancement of the gut barrier function (Corr et al., 2007; Walsh et al., 2008; Kommineni et al., 2015; Umu et al., 2016; Heeney et al., 2019). Gonzalez and Kunka (1987) reported that a 6.2-megadalton plasmid was responsible for the production of bacteriocin in *P*. *acidilactici* PAC1.0, designated as pediocin PA-1, which had a molecular weight of ca. 16,500. Subsequently, a variety of bacteriocins have been discovered and identified, including pediocin Ach, pediocin JD1, pediocin SJ1, and pediocin N5P. At present, *P*. *acidilactici* strains are widely used in the food industry, animal husbandry, and medical settings. The bacteriocins produced are known to inhibit the growth of pathogenic microorganisms in the host or to act as signal-regulated peptides to regulate the health of the host (Chelliah et al., 2020). Although many studies have confirmed the promising effects of several probiotics in the treatment of constipation, the degree of alleviation provided by bacteriocin-producing *P*. *acidilactici* strains and their regulatory roles on the intestinal flora remain poorly understood. Therefore, it is necessary to investigate the effects of bacteriocin-producing and non-bacteriocin-producing *P*. *acidilactici* strains on constipation-related symptoms and the composition of the intestinal flora.

In this study, loperamide hydrochloride was used to induce constipation in mice, which were then treated with different *P*. *acidilactici* strains. The constipation-related symptoms were identified to evaluate the effects of different strains on the constipation. Besides, to explore the underlying mechanism of the effect of the *P*. *acidilactici* strains on constipation, an enzyme-linked immunosorbent assay and a high-throughput MiSeq sequencing technique were used to analyze the levels of gastrointestinal regulatory peptides and the composition of the fecal microbiota, respectively.

## Materials and Methods

### Chemicals and Reagents

The levels of motilin (MTL), gastrin (Gas), substance P (SP), endothelin (ET), somatostatin (SS) and vasoactive intestinal peptide (VIP) were measured using a kit purchased from Wen LE Bioengineering Institute (Shanghai, China). MRS broth was obtained from Qingdao Hope Bio-Technology Co., Ltd (Qingdao, China). Loperamide hydrochloride (2 mg per capsule) was obtained from Wuxi Pharmacy (Xi’an Janssen Pharmaceutical Ltd., Xi’an, China). Loperamide hydrochloride was dissolved in distilled water to a final concentration of 1 mg/mL. Phenolphthalein (0.1 g, 100 tablets) was dissolved in distilled water to a final concentration of 7 mg/mL.

For the activated carbon meal solution, we added 100 g of gum arabic to 800 mL of water, which was boiled until the solution became transparent. We then added 50 g of activated carbon to the solution and boiled it three times. After the solution had cooled to room temperature, we added water to adjust the volume to 1000 mL, stored it at 4°C, and mixed it prior to use. All other chemicals and reagents used in this study were of analytical grade.

### Bacterial Strains and Growth Media

The bacteriocin-producing *P*. *acidilactici* CCFM28 strain was provided by the University of Groningen in the Netherlands, and *P*. *acidilactici* CCFM 18 strain was isolated from Chinese pickles. The non-bacteriocin-producing *P*. *acidilactici* NT17-3 and 102H8 strains were isolated from human feces.

To activate and amplify the *P*. *acidilactici* strains, an MRS medium was used that contained 10.0 g of peptone, 5.0 g of beef extract, 4.0 g of yeast extract, 20.0 g of glucose, 2.0 g of K_2_HPO_4_, 2.0 g of ammonium citrate, 5.0 g of sodium acetate, 0.2 g of MgSO_4_, 0.05 g of MnSO_4_, and 1.0 mL of Tween 80 per liter. After adjusting the pH to 6.2 ± 0.2, we sterilized the medium at 115°C for 20 min and added 15.0 g/L of agar to obtain a solid medium.

Four strains (including two bacteriocin-producing and two non-bacteriocin-producing strains) were used for intragastric administration to the constipated mice. Briefly, the *P*. *acidilactici* strains were activated at 37°C for 48 h, and the bacterial suspensions were then centrifuged at 5,000 g for 15 min to generate deposits. After washing with phosphate-buffered saline three times, the clean bacterial deposits were dissolved in sterile skim milk solution, followed by lyophilization into a powder. The freeze-dried samples were then resuspended in physiological saline and adjusted to 10^9^ CFU/mL for administration to the mice.

### Animals and Sample Collection

Seven-week-old female BALB/c mice, provided by the Shanghai Laboratory Animal Center (Shanghai, China), were kept in a standard rodent cage at a constant temperature and humidity under a strict 12-h light cycle. The mice were fed a commercial mouse diet and drinking water was freely available.

After being allowed to adapt to their new environment for one week, the mice were randomly divided into seven groups (n = 10 for each group) (Battish et al., 2000). Apart from the normal control group, loperamide hydrochloride (10 mg/kg, 200 μL) was intragastrically administered daily to the mice in the other six groups for 14 d. Both the constipated and normal mice were fed a commercial diet and freely available drinking water. The mice in the *P*. *acidilactici*-treated group were given 200 μL of bacterial suspension *via* intragastric infusion each day, including the bacteriocin-producing *P*. *acidilactici* CCFM28 and CCFM18 strains, and non-bacteriocin-producing *P*. *acidilactici* NT17-3 and 102H8 strains. In contrast, 200 μL of physiological saline and phenolphthalein (70 mg/kg body weight) were given to the negative and positive control groups, respectively. The control groups were fed the control diet (FOS, 0%) throughout the experiment (Lin et al., 2005). The body weights of the mice in all groups were recorded each day and their feces were collected daily for examination. After 14 d of treatment, the mice in all groups were fasted for 12 h, although water was still available. Eyeball blood was then extracted to measure the level of gastrointestinal regulation peptides. After sacrificing the mice *via* cervical dislocation, the entire intestines were removed to measure the rate of advancement of ink through the small intestine. The contents of the colon and cecum were also collected and taken to the laboratory within 2 h for extraction of the intestinal genome.

### Detection of Constipation-Related Indicators

To analyze the effects of different *P*. *acidilactici* strains on constipation in mice, we compared the time to the first black-stool defecation, using the method reported by Lee et al. (2012). Briefly, a mixture of ink and the corresponding test substances were given to the mice that had been constipated by loperamide hydrochloride, and the time to the first black-stool defecation for each mouse was recorded until the first black stools of all mice in the negative control group had been excreted.

The gastrointestinal transit rate provides information about intestinal motility and constipation-related symptoms. After the constipated mice had been intragastrically infused with a mixture of ink and the corresponding test substances for 30 min, they were sacrificed *via* cervical dislocation. The gastrointestinal transit rate was calculated as follows (Nagakura et al., 1996):

$$Gastrointestinal\;transit\;rate\text{(\%) }=\frac{Ink\;advancement\;length}{The\;total\;length\;of\;the\;small\;intestine}\times 100\%$$

### Analysis of Intestinal Microflora

The FastDNA Spin Kit for Soil (MP Biomedicals, California, U.S.A.) was used to extract the microbial genomic DNA from the collected fecal samples. After quantifying the obtained DNA, a polymerase chain reaction (PCR) analysis of the V4 variable region of the bacterial 16S rRNA was performed using the primers (forward primer, 5′-CCTAYGGGRBGCASCAG-3′; reverse primer, 5′-GGACTACNNGGGTATCTAAT-3′). The obtained PCR products were separated by electrophoresis in agarose gels (2%, w/v), followed by purification by the Qiagen Gel Extraction Kit (Qiagen, Germany) and incorporation at equal concentrations (Qiu et al., 2018b). Lastly, the PCR-purified products were sequenced on an Illumina MiSeq 250 platform (Illumina, San Diego, CA, USA) according to the protocol described by Caporaso et al. (2010).

### Bioinformatic Analysis

Using the QIIME program (http://qiime.sourceforge.net/), quality raw data were selected, in which sequences with an average length < 2000 bp or an average quality score < 25 were removed (Caporaso et al., 2010). In addition, chimera sequences were discarded using the UCHIME algorithm (Edgar et al., 2011). Using fast length adjustment of short reads (FLASH, v1.2.7), we assigned paired-end reads to each sample based on their unique barcodes and arranged them according to their overlapping sequences (Magoč and Salzberg, 2011). After trimming the barcode and sequence primers from the sequences, the valid sequences were classified into operational taxonomic units (OTUs) based on the sequence similarity with a 0.97 similarity threshold (Qiu et al., 2018a). Using the Naive Bayes classifier of the Ribosomal Database Project (RDP), the SILVA SSU database at the genus, family, order, class, and phylum levels was used to classify and annotate the taxonomic information for each representative OTU sequence (Quast et al., 2012; Yilmaz et al., 2014; Kalyuzhnaya et al., 2015). Using QIIME (Version 1.7.0), the alpha diversity was calculated to determine the diversity of the microbial community, and R software (Version 2.15.3) was used to analyze the beta diversity and compare the differences in the different treatment groups.

## Results

### Effect of *P*. *acidilactici* Strains on Time to First Black-Stool Defecation

**Figure 1:**
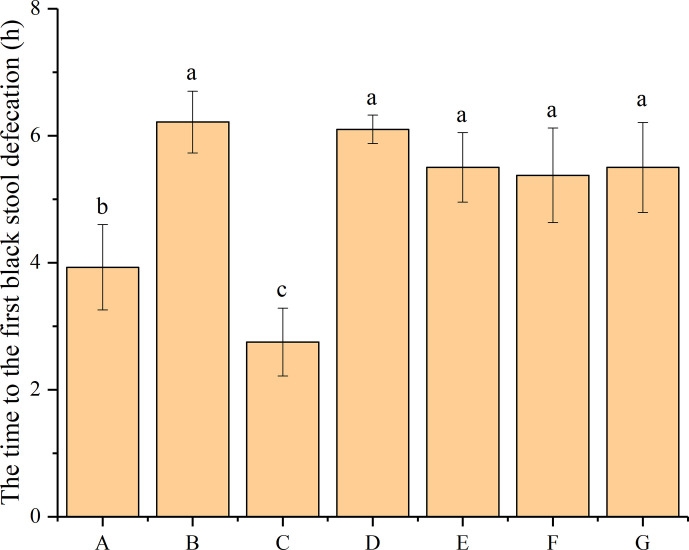
Time to the first black-stool defecation in BALB/c mice after constipation had been induced by loperamide hydrochloride and different treatments were given (n = 10). **(A)** Normal control: no treatment; **(B)** negative control group: physiological saline; **(C)** positive control group: phenolphthalein (70 mg/kg body weight); **(D)** treatment group: *P*. *acidilactici* CCFM28 strain; **(E)** treatment group: *P*. *acidilactici* CCFM18 strain; **(F)** treatment group: *P*. *acidilactici* NT17-3 strain; **(G)** treatment group: *P*. *acidilactici* 102H8 strain. The letters a, b, and c indicate significant differences (P < 0.05) as determined using Duncan’s multiple range test.

The time to the first black-stool defecation was compared to evaluate the effect of bacteriocin-producing and non-bacteriocin-producing *P*. *acidilactici* strains on constipation in mice. As shown in **Figure 1**, the time to the first black-stool defecation of the different groups ranged from 3.92 h to 6.21 h, with obvious differences. The time to the first black-stool defecation of mice in the normal group was 3.93 h, which significantly increased to 6.21 h after treatment with loperamide hydrochloride (*P* < 0.05). However, the administration of phenolphthalein and four *P*. *acidilactici* strains was found to reduce the time to the first black-stool defecation in the mice. The shortest time to the first black-stool defecation occurred in the mice treated with phenolphthalein (2.75 h), which differed significantly from that of the normal group (*P* < 0.05). Although the time to the first black-stool defecation of mice in the *P*. *acidilactici* -treated groups was shorter than that of the negative control group, the differences were not significant (*P* > 0.05). Among the four strain-treatment groups, the mice treated with *P*. *acidilactici* NT17-3 had the shortest time to the first black-stool defecation (5.38 h), and the mice treated with *P*. *acidilactici* CCFM28 had the longest time (6.10 h). This time was 5.50 h for mice treated with the *P*. *acidilactici* CCFM18 and 102H8 strains. These results indicated that both the bacteriocin-producing and non-bacteriocin-producing *P*. *acidilactici* strains had limited therapeutic effects on constipation-related symptoms in mice. Interestingly, although there were no significant differences between the time to the first black-stool defecation in mice from the four *P*. *acidilactici*-treated groups, the non-bacteriocin-producing *P*. *acidilactici* strains seemed to contribute to a decreased time of first defecation.

### Effect of *P*. *acidilactici* Strains on the Gastrointestinal Transit Rate

**Figure 2:**
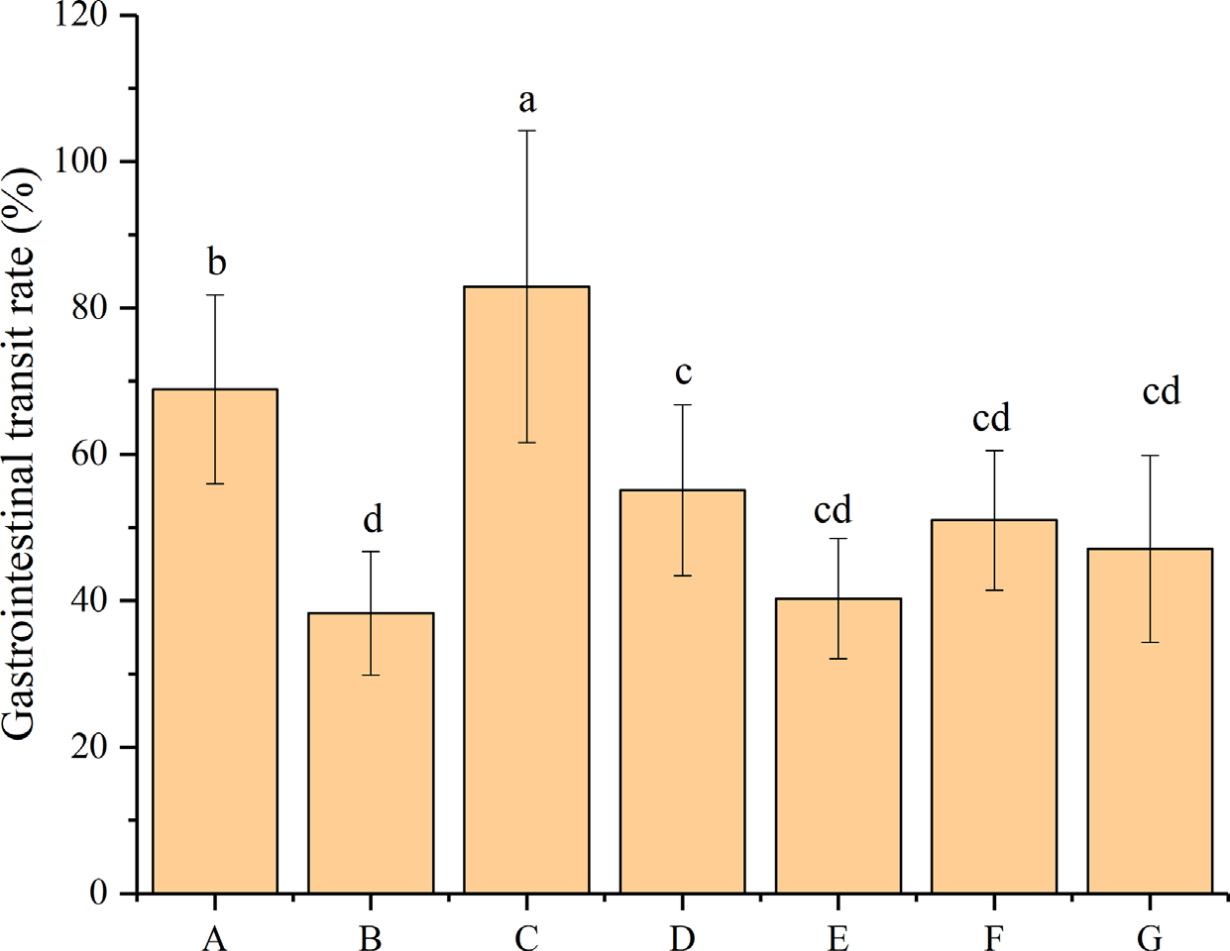
Gastrointestinal transit rate of BALB/c mice after constipation induced by loperamide hydrochloride and administration of different treatments (n = 10). **(A)** Normal control: no treatment; **(B)** negative control group: physiological saline; **(C)** positive control group: phenolphthalein (70 mg/kg body weight); **(D)** treatment group: *P*. *acidilactici* CCFM28 strain; **(E)** treatment group: *P*. *acidilactici* CCFM18 strain; **(F)** treatment group: *P*. *acidilactici* NT17-3 strain; **(G)** treatment group: *P*. *acidilactici* 102H8 strain. The letters a, b, c and d indicate significant differences (P < 0.05) as determined using Duncan’s multiple range test.

The gastrointestinal transit rate indicates the peristalsis or movement through the small intestine during digestion. According to the results shown in **Figure 2**, the administration of loperamide hydrochloride resulted in a 44.41% decrease in the gastrointestinal transit rate (from 68.90% to 38.30%), which is significantly different from that of the mice in the normal control group (*P* < 0.05). This result indicated the successful establishment of a constipated model with the obstruction of normal peristalsis of the small intestine. The mice subsequently treated with phenolphthalein exhibited the highest gastrointestinal transit rate (83%), which was significantly higher than those of the mice in other groups (*P* < 0.05). The administration of four *P*. *acidilactici* strains resulted in different increases in their gastrointestinal transit rates and improvements in their constipation-related symptoms. Mice treated with bacteriocin-producing *P*. *acidilactici* CCFM28 strains exhibited an increase from 38.30% to 55.10% in the gastrointestinal transit rate, which differed significantly from that of the negative control group (*P* < 0.05). However, although the other three *P*. *acidilactici* strains increased the gastrointestinal transit rate in the mice, the differences between the *P*. *acidilactici*-treated and negative control groups were not significant (*P* > 0.05). We noted that the mice treated with the bacteriocin-producing *P*. *acidilactici* CCFM18 strain showed the lowest gastrointestinal transit rate (40.30%). Furthermore, the recovery of the gastrointestinal transit function of the mice treated with different *P*. *acidilactici* strains did not reach normal levels.

Based on the results obtained from the time to the first black-stool defecation and the gastrointestinal transit rate, the four *P*. *acidilactici* strains exhibited limited improvement in the constipation-related symptoms of the mice. The non-bacteriocin-producing *P*. *acidilactici* NT17-3 strain had the best effect on the time to the first black-stool defecation, and the bacteriocin-producing *P*. *acidilactici* CCFM28 strain significantly improved the small-intestine peristalsis.

### Effect of *P*. *acidilactici* Strains on Gastrointestinal Regulatory Peptides in Serum of Constipated Mice

Gastrointestinal regulatory peptides related to constipation, such as SP, MTL, Gas, ET, SS, and VIP, play crucial roles in the regulation of gastrointestinal motility (Schulz et al., 2004). Among them, three peptides (SP, MTL, and Gas) are known to be excitatory neurotransmitters, with the other three peptides (SS, VIP, and ET) being inhibitory neurotransmitters. SP, a neuropeptide that is widely distributed in fine nerve fibers, is reported to enhance the contraction of gastrointestinal smooth muscles and promote gastrointestinal motility (Du et al., 2003). Similarly, gastrointestinal motility is improved by MTL and Gas *via* the regulation of muscular movement and the secretion and transportation of digestive juices. In contrast, SS and VIP are considered to be regulatory inhibitory peptides that inhibit the secretion of several digestive enzymes and movement of intestinal muscles (Lucey, 1986).

**Figure 3:**
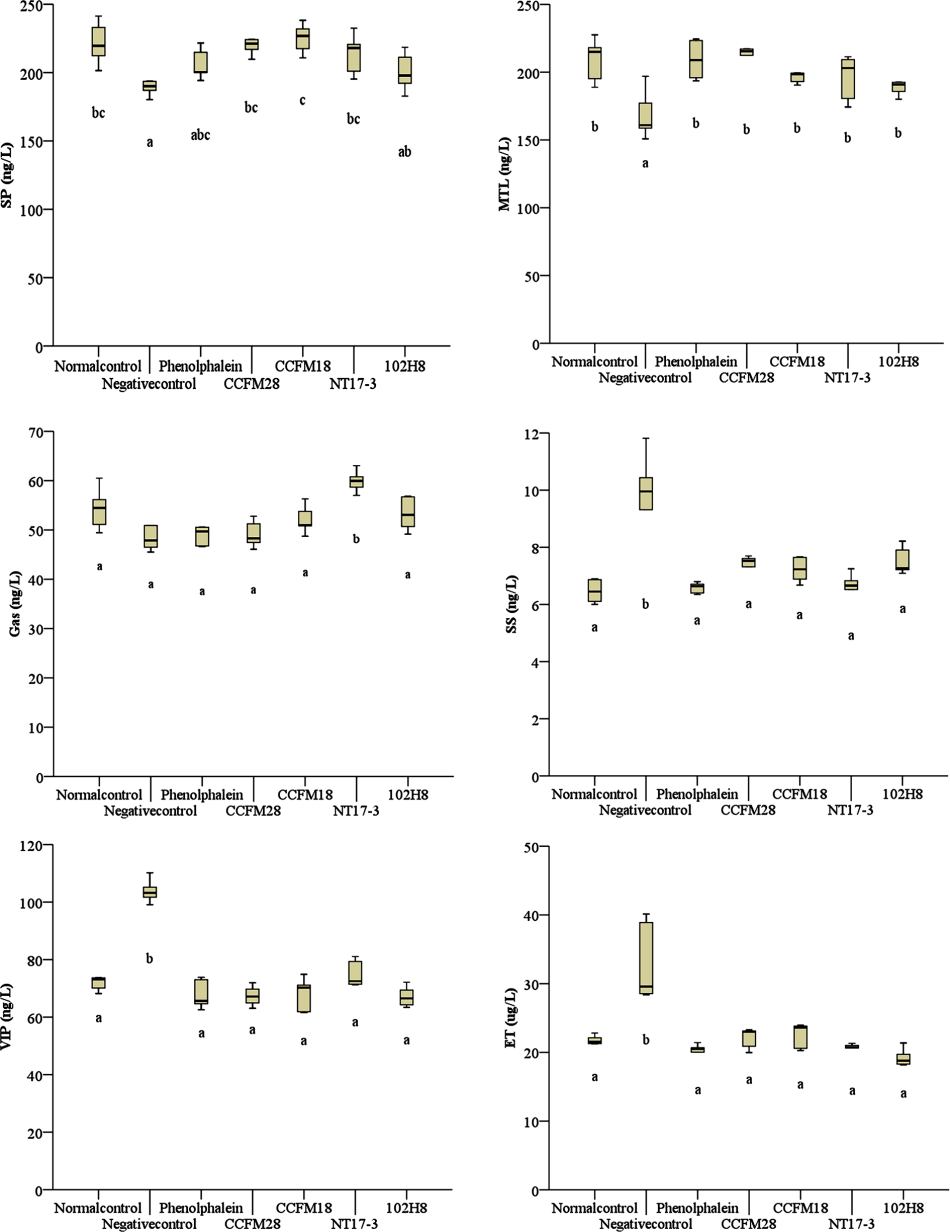
Levels of gastrointestinal regulatory peptides in the serum of BALB/c mice after constipation had been induced by loperamide hydrochloride and different treatments were given (n = 10). SP: substance P; MTL: motilin; Gas: gastrin; SS: somatostatin; VIP: vasoactive intestinal peptide; ET: endothelin. **(A)** Normal control: no treatment; **(B)** negative control group: physiological saline; **(C)** positive control group: phenolphthalein (70 mg/kg body weight); **(D)** treatment group: *P*. *acidilactici* CCFM28 strain; **(E)** treatment group: *P*. *acidilactici* CCFM18 strain; **(F)** treatment group: *P*. *acidilactici* NT17-3 strain; **(G)** treatment group: *P*. *acidilactici* 102H8 strain. The letters a, b, and c indicate significant differences (P < 0.05) as determined using Duncan’s multiple range test.

As shown in **Figure 3**, the excitatory neurotransmitters (SP, MTL, and Gas) in the mice treated with loperamide hydrochloride exhibit a downward trend, whereas the inhibitory neurotransmitters (SS, VIP, and ET) were significantly upregulated (*P* < 0.05). This indicated that the ingestion of loperamide hydrochloride resulted in decreased gastrointestinal motility and mucus secretion. The phenolphthalein and four *P*. *acidilactici* strains effectively regulated the levels of excitatory and inhibitory neurotransmitters, including the upregulation of SP, MTL, and Gas, and the downregulation of SS, VIP, and ET. Specifically, the MTL level was significantly increased by 16.18–27.53% following the administration of *P*. *acidilactici* strains (*P* < 0.05), compared with the negative control group. Regarding SP and Gas, treatment with the four *P*. *acidilactici* strains resulted in 4.82–17.65% and 1.73–23.89% increases, respectively. Moreover, only the non-bacteriocin-producing *P*. *acidilactici* NT17-3 strain caused a significant increase in Gas (*P* < 0.05), whereas only the increase in SP was not significant in the mice treated with the non-bacteriocin-producing *P*. *acidilactici* 102H8 strain (*P* < 0.05).

In contrast, all three inhibitory neurotransmitters (SS, VIP, and ET) were downregulated after the introduction of different *P*. *acidilactici* strains, with the degree of reduction showing a significant difference (*P* < 0.05). The SS level decreased by 22.57–31.88% following treatment with the *P*. *acidilactici* strains, with the ET level decreasing by 32.19–41.78% and the VIP level decreasing by 27.67–35.35%. We noted that the *P*. *acidilactici* strains showed greater regulatory effects on the inhibitory neurotransmitters than on the excitatory neurotransmitters. Furthermore, significant differences between different *P*. *acidilactici* strains occurred only in Gas between the *P*. *acidilactici* NT17-3 strain and three other strains, and in SP between the *P*. *acidilactici* NT17-3 and 102H8 strains.

### Sequence and OTU Statistical Analysis

Using the Illumina MiSeq sequencing platform, a total of 929,810 high-quality 16S rRNA gene sequences were generated from 70 fecal samples. For each sample, the average sequence read was 13,283 after sequence assembly and quality control. At a 97% confidence threshold, all of the sequences were clustered with the representative sequences, and the number of OTUs ranged from 8,639 to 63,803.

### Effect of *P*. *acidilactici* Strains on the Alpha and Beta Diversities of Fecal Microbiota

The alpha diversity index reflects the abundance and diversity of the microbial community in an individual sample, including coverage, the chao1 index, observed species, Shannon index, and PD_whole_tree. As shown in **Table 1**, the five index values of the normal group averaged 0.9151, 5076.11, 1079.0, 5.60, and 72.93, respectively. The administration of phenolphthalein in the constipated mice led to great changes in these indexes to 0.9287, 7599.74, 1668.5, 5.32, and 94.31, respectively. For the *P*. *acidilactici*-treated groups, the application of different strains resulted in an increasing trend of the alpha diversity of the microbial community in the fecal microbiota. Specifically, although there was no significant difference in the Shannon indices of the seven groups, the chao1 and observed species indices of the mice treated with the bacteriocin-producing *P*. *acidilactici* CCFM28 strain were significantly higher than those of the other groups. This result indicated that this strain significantly increased the microbial diversity of the intestinal flora of the mice (*P* < 0.05).

**Table 1:** Alpha diversity indexes of microbial communities.

Groups	Coverage	Chao1 index	Observed species	Shannon index	PD_whole_tree
Normal control group	0.9151^b^	5076.11^d^	1079.0^d^	5.60^a^	72.93^c^
Negative control group	0.9292^ab^	10470.77^bc^	2432.2^bc^	5.94^a^	124.50^b^
Phenolphthalein	0.9287^ab^	7599.74^cd^	1668.5^cd^	5.32^a^	94.31^bc^
CCFM28	0.9455^a^	16291.02^a^	3876.5^a^	5.73^a^	179.68^a^
CCFM18	0.9196^b^	4924.21^d^	1165.5^d^	5.60^a^	78.30^c^
NT17-3	0.9447^a^	12021.24^b^	2781.3^b^	5.72^a^	137.92^b^
102H8	0.9318^ab^	8989.47^bcd^	1970.6^bcd^	5.56^a^	105.32^bc^

Different small letters in the same column means a significant difference (P < 0.05).

**Figure 4:**
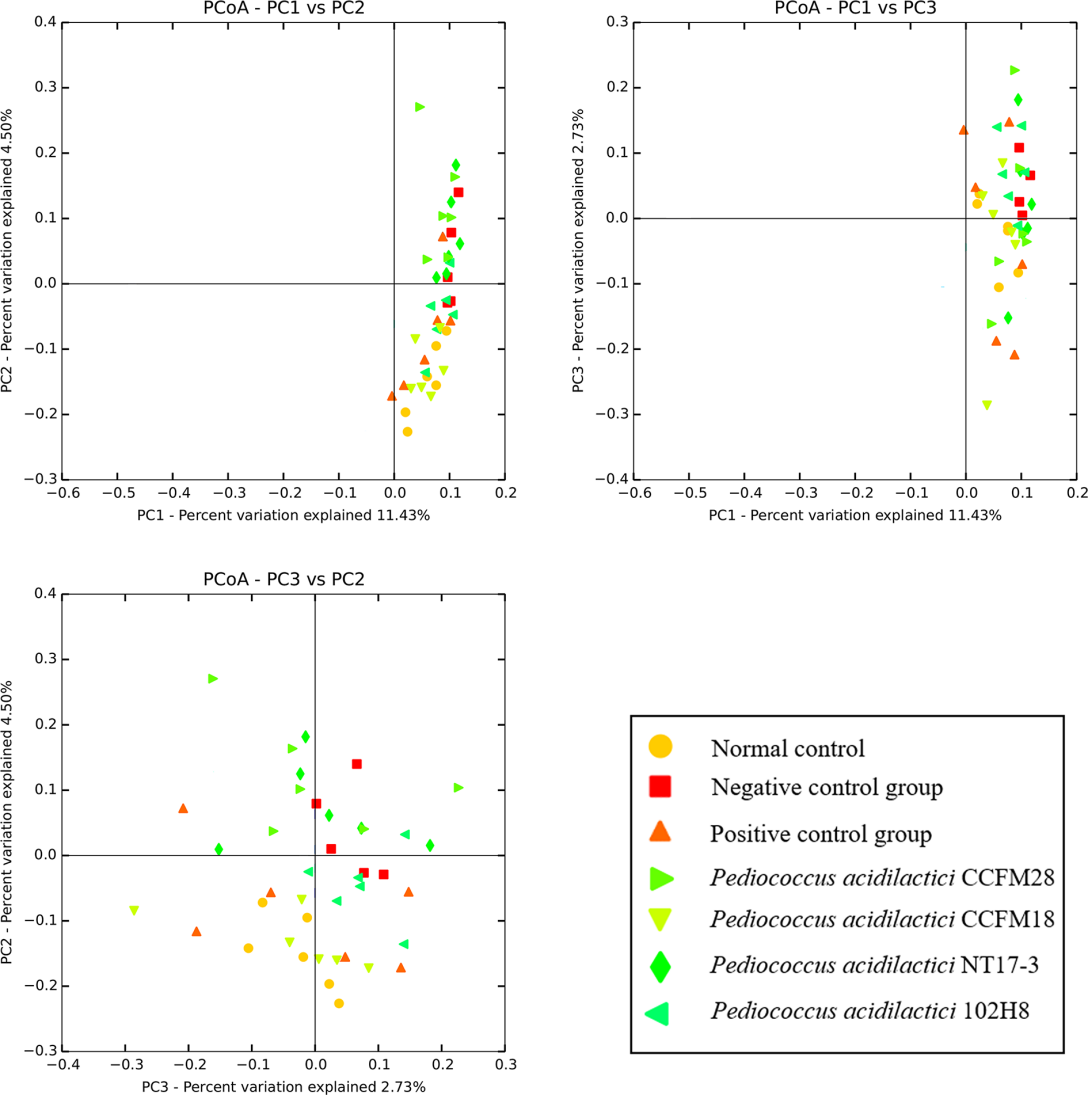
Principal component analysis of microbial communities in mice feces.

**Figure 4** showed an unweighted uniFrac matrix of the beta diversities in the gut microbiota of mice treated with the four *P*. *acidilactici* strains at the genus level, which revealed differences and similarities among the samples. The distribution maps of the first two principal components represented 11.43% (PC1) and 4.5% (PC2) of the accumulative variance contributions, for a total of 15.9%. The microbial communities of the mice treated with phenolphthalein and the mice in the normal control group were clustered together, which indicated that treatment with phenolphthalein was effective in adjusting the structure of the intestinal flora in constipated mice to a normal level. The microbial communities of mice treated with the *P*. *acidilactici* CCFM18 and 102H8 strains and the mice in the normal control group also overlapped to some extent, which indicated a high degree of similarity in their microbial communities. However, the microbial communities of the mice treated with the *P*. *acidilactici* CCFM28 and NT17-3 strains could be readily distinguished from those of the normal control group, which meant that the application of *P*. *acidilactici* NT17-3 clearly changed the structure of the gut microbiota in the mice.

### Effect of *P*. *acidilactici* Strains on the Composition of Fecal Microbiota at the Phylum Level

**Figure 5:**
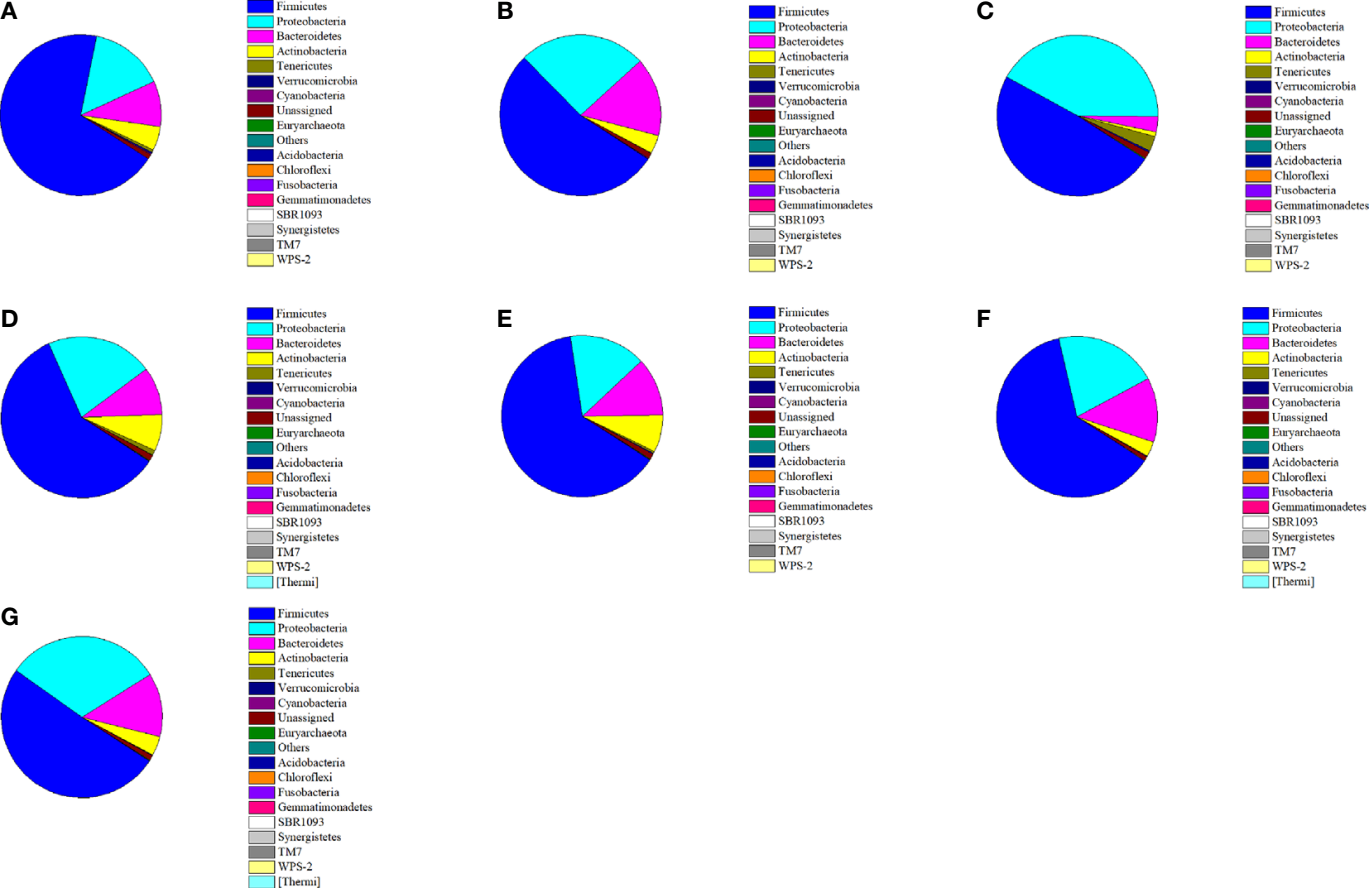
Relative abundance of main phyla in the different mice groups after constipation induced by loperamide hydrochloride and the administration of different treatments (n = 10). **(A)** Normal control: no treatment; **(B)** negative control group: physiological saline; **(C)** positive control group: phenolphthalein (70 mg/kg body weight); **(D)** treatment group: *P*. *acidilactici* CCFM28 strain; **(E)** treatment group: *P*. *acidilactici* CCFM18 strain; **(F)** treatment group: *P*. *acidilactici* NT17-3 strain; **(G)** treatment group: *P*. *acidilactici* 102H8 strain.

**Figure 5** showed the distributions and variances in the bacterial community compositions at the phylum level in normal and constipated mice given different treatments. The fecal flora of normal mice mainly consisted of Firmicutes (68.98%), Proteobacteria (15.00%), and Bacteroidetes (9.23%). Other phyla were also detected, but they were low in abundance (< 1%), including Tenericutes, Verrucomicrobia, and Cyanobacteria. After treatment with loperamide hydrochloride, great changes occurred in the fecal microbiomes of mice in the negative control group. The relative abundance of Firmicutes was significantly reduced by 29.21% (from 68.98% to 53.54%), whereas the relative abundances of Proteobacteria and Bacteroidetes were significantly increased by 70.33% and 71.55%, respectively (*P* < 0.05). The administration of the four *P*. *acidilactici* strains effectively reversed this change, where the relative abundances of Firmicutes and Actinobacteria were upregulated, and those of Proteobacteria and Bacteroidetes were downregulated. The Firmicutes included some intestinal probiotics (i.e., Lactobacillus and Lactococcus), and the Proteobacteria contained many pathogens (including Salmonella and Escherichia coli). Therefore, changes derived from the interference of the *P*. *acidilactici* strains indicated the improvement in the intestinal environment.

The distribution and relative abundance of the bacterial community at the phylum level of the mice in the *P*. *acidilactici* CCFM28-treated group were closest to those of mice in the normal group. The relative abundance of Firmicutes increased from 53.54% to 63.53%, but the Proteobacteria and Bacteroidetes decreased from 25.54% and 15.84% to 15.38% and 11.74%, respectively. The microbial community of the mice in the *P*. *acidilactici* CCFM18-treated group exhibited changes similar to those of the *P*. *acidilactici* CCFM28-treated group, which indicated that it also effectively regulated the composition of the mouse intestinal flora. An increase of 10.52% in the relative abundance of Firmicutes and decreases of 16.00% and 38.78% in the respective levels of Proteobacteria and Bacteroidetes occurred in the mice treated with the *P*. *acidilactici* CCFM18 strain. In the non-bacteriocin-producing *P*. *acidilactici*-treated groups, the regulation of intestinal flora by the *P*. *acidilactici* NT17-3 strain was similar to that of the bacteriocin-producing *P*. *acidilactici* CCFM28 strain, including an increase of 16.10% in Firmicutes and decreases of 18.39% and 18.66% in Proteobacteria and Bacteroidetes, respectively. Although the phyla with high abundance in the *P*. *acidilactici* 102H8-treated group were almost identical to those of the other groups, there was no obvious change in the relative abundance of Firmicutes. Therefore, we could conclude that all four *P*. *acidilactici* strains reduced the relative abundances of Proteobacteria and Bacteroidetes and increased the level of Firmicutes, but *P*. *acidilactici* CCFM28 was most effective.

The observed changes in the intestinal flora were similar to the results reported by Wang et al. (2017). They found that the intervention of *Bifidobacterium adolescentis* CCFM 669 and 667 strains increased the ratio of relative abundance of Firmicutes to Bacteroidetes at the phylum level in constipated BALB/c mice (Wang et al., 2017). In our study, the administration of loperamide hydrochloride in mice was found to disturb the ecological balance of intestinal flora, which was mainly manifested in the reduced levels of Firmicutes and the increased levels of Proteobacteria and Bacteroidetes (including many pathogens or enteropathogenic bacteria). However, the disruption of the microbial structure was moderated by the introduction of four *P*. *acidilactici* strains, which might generate a space-occupying competition or the production of some metabolites (such as bacteriocins and SCFAs).

### Effect of *P*. *acidilactici* Strains on the Composition of Fecal Microbiota at the Genus Level

**Figure 6:**
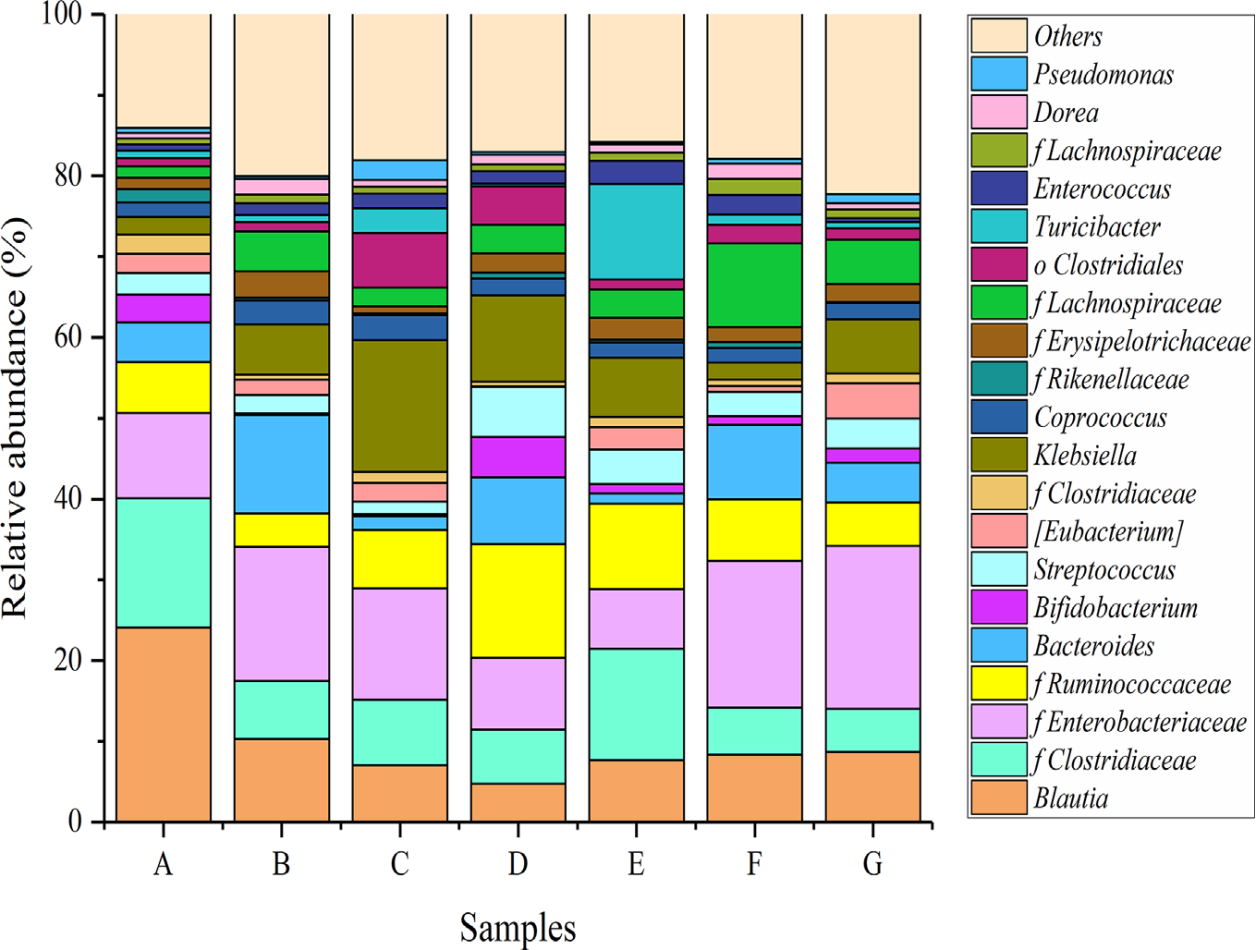
Relative abundances of main genera in the different mice groups after constipation had been induced by loperamide hydrochloride and different treatments were administered (n = 10). **(A)** Normal control: no treatment; **(B)** negative control group: physiological saline; **(C)** positive control group: phenolphthalein (70 mg/kg body weight); **(D)** treatment group: *P*. *acidilactici* CCFM28 strain; **(E)** treatment group: *P*. *acidilactici* CCFM18 strain; **(F)** treatment group: *P*. *acidilactici* NT17-3 strain; **(G)** treatment group: *P*. *acidilactici* 102H8 strain.

At the genus level, a total of 675 bacterial genera were classified, of which 22 genera were found to have relative abundances greater than 1%. **Figure 6** showed the relative abundances of main genera in the different mice groups. The relative abundance of *Blautia* was greatest in the mice of the normal control group (24.10%), followed by several genera from *Clostridiaceae* (16.01%), *Enterobacteriaceae* (10.53%), and *Ruminococcaceae* (6.30%). *Bacteroides* and *Bifidobacterium* also accounted for large proportions (4.94% and 3.43%, respectively). Treatment with loperamide hydrochloride resulted in a major change in the relative abundance of the bacterial community in mice. The proportions of *Blautia*, *Bifidobacterium*, *Lactobacillus*, and some genera from *Clostridiaceae* and *Ruminococcaceae* dropped significantly, whereas *Bacteroides*, *Klebsiella*, and certain genera from *Enterobacteriaceae* were obviously increased.

After the administration of *P*. *acidilactici* strains, the relative abundances of *Bifidobacterium* and *Lactobacillus* increased significantly (*P* < 0.05), i.e., 4–21-fold and 1–37-fold, respectively, compared with those in the negative control group. *Bifidobacterium* and *Lactobacillus* are considered to be important intestinal beneficial microorganisms that can regulate gastrointestinal function and enhance human immunity (Meile et al., 2008). A significant decrease in the relative abundance of *Bacteroides* was also observed following treatment with the four *P*. *acidilactici* strains, with a 24.13–89.34% decrease compared with the negative control group. The authors of another study reported that *Bacteroides* inhabited the human intestines and might cause endogenous infections when the body’s immune function was disrupted or the flora was unbalanced due to the use of antibiotics. The relative abundances of *Blautia* and several genera from *Erysipelotrichaceae* and *Lachnospiraceae* further decreased when the *P*. *acidilactici* strains were administered. *Enterobacteriaceae* contains a variety of intestinal pathogens, such as *Escherichia coli*, *Salmonella*, and *Shigella*, which are closely associated with intestinal diseases (including bacterial diarrhea, gastroenteritis, and dysentery) (Coburn et al., 2007).

Compared with the negative control group, treatment with the bacteriocin-producing *P*. *acidilactici* CCFM28 and CCFM18 strains significantly decreased the relative abundance of the genera from *Enterobacteriaceae*, especially the *P*. *acidilactici* CCFM28 strain. However, non-bacteriocin-producing *P*. *acidilactici* strains did not achieve this effect, which might be due to the absence of bacteriocin.

## Discussion

Constipation is a common gastrointestinal functional disorder worldwide and seriously affects the quality of life of patients. The effectiveness of probiotics in relieving functional constipation is reported to be due to their regulation of aberrant gut motility *via* metabolites such as SCFAs (Dimidi et al., 2017) and tryptamine (Bhattarai et al., 2018). However, there have been no reports regarding differences in the degree of alleviation provided by different *P*. *acidilactici* strains (bacteriocin-producing and non-bacteriocin-producing) of constipation or the resulting changes in the fecal flora in mice.

The results of this study indicated that the administration of four *P*. *acidilactici* strains produced some improvement in constipation-related symptoms (based on the time to the first black-stool defecation and gastrointestinal transit rate), but the difference was not significant (*P* > 0.05). However, there were significant differences in the compositions and relative abundances of the intestinal microbiota in different groups. At the phylum level, the relative abundance of Firmicutes significantly increased, but those of Bacteroidetes and Proteobacteria decreased after the administration of four *P*. *acidilactici* strains for 14 d. Furthermore, the levels of *Bacteroides* and several genera from *Enterobacteriaceae* were significantly decreased, whereas *Bifidobacterium* and *Lactobacillus* were upregulated following the provision of bacteriocin-producing *P*. *acidilactici* CCFM18 and CCFM28 strains in the diet. Therefore, although constipation-related symptoms were only marginally alleviated, the four *P*. *acidilactici* strains were found to regulate the intestinal flora and provide a potential health benefit to the host. Bacteriocin-producing *P*. *acidilactici* strains, in particular, were more effective in restoring the intestinal flora to normal levels.

Bacteriocins, as ribosomally synthesized peptides or proteins with potential antimicrobial activity, are produced by many bacterial species (Gálvez et al., 2007). Bacteriocin production may play an important role in the competition within complex microbial communities or in positively influencing the health of the host. The factors that contribute to its probiotic functionality may derive from several mechanisms within the gastrointestinal tract. For example, some bacteriocins have been considered to be colonizing peptides that can facilitate the introduction of a probiotic into an existing niche or compete with the resident microbiota (Riley and Wertz, 2002). Bacteriocins may also function as antimicrobial peptides that can inhibit or eliminate pathogens (Majeed et al., 2011). Some signaling peptides may signal the immune cells of the host or other bacteria (Di Cagno et al., 2007; Gobbetti et al., 2007). These mechanisms may act alone or in combination in the gastrointestinal tract of mice supplemented with bacteriocin-producing *P*. *acidilactici* CCFM18 and CCFM28 strains. However, additional research is required to clarify how those two strains affect intestinal flora.

The administration of *P*. *acidilactici* strains resulted in significant increases in the relative abundances of *Bifidobacterium* and *Lactobacillus* (4–21-fold and 1–37-fold, respectively), compared with that in the negative control group (*P* < 0.05). Furthermore, administration of the bacteriocin-producing *P*. *acidilactici* CCFM28 and CCFM18 strains significantly decreased the relative abundance of the genera from *Enterobacteriaceae*. The increase in beneficial bacteria and decrease in harmful bacteria are closely related to improvement of the constipation symptoms. For example, the SCFAs produced by *Bifidobacterium* and *Lactobacillus* were found to relieve symptoms of constipation by stimulating the absorption of water and electrolytes, promoting the proliferation of epithelial cells, improving gastrointestinal motility, and increasing mesenteric blood flow (Ríos-Covián et al., 2016). Furthermore, in this study, the four *P*. *acidilactici* strains were found to cause upregulation of excitatory neurotransmitters (SP, MTL, and Gas) and the downregulation of inhibitory neurotransmitters (SS, VIP, and ET). Therefore, changes in the gut bacteria can also alleviate constipation by influencing the levels of gastrointestinal hormones, which counteract the symptoms of constipation *via* the enhancement of intestinal peristalsis and the transport of contents. These findings agree well with the results of Wang et al. (2017).

Extensive use of antibiotics might increase the relative abundance of *Clostridium*, leading to antibiotic-related diarrhea and pseudomembranous colitis (Kim et al., 1981). Treatment with bacteriocin-producing *P*. *acidilactici* CCFM18 strain resulted in a 6.34% decrease in the relative abundance of the genera from *Clostridiaceae*, thereby reducing the likely incidence of inflammatory diseases. Therefore, the decrease in the relative abundance of harmful intestinal microorganisms and the increase in probiotics indicated that the administration of bacteriocin-producing and non-bacteriocin-producing *P*. *acidilactici* strains had the potential to regulate the intestinal flora, maintain human health, and replace antibiotic drugs, with the bacteriocin-producing *P*. *acidilactici* CCFM28 strain being especially effective.

## Conclusions

In conclusion, we investigated the effects of the administration of bacteriocin-producing and non-bacteriocin-producing *Pediococcus acidilactici* strains on constipation symptoms, gastrointestinal regulatory peptides and gut microbiota in BALB/c mice. The treatment of four *P*. *acidilactici* strains could limitedly improve the first black-stool defecation and the gastrointestinal transit rate in constipated mice (*P* > 0.05), significantly regulated the level of most gastrointestinal regulatory peptides in the serum (*P* < 0.05). Furthermore, the composition and abundance of intestinal microbiota in different groups were greatly changed by four *P*. *acidilactici* strains. The intervention of four *P*. *acidilactici* strains on constipated mice effectively reversed the change in the gut flora caused by the treatment of loperamide hydrochloride and restored their composition and relative abundance. Therefore, although constipation-related symptoms were alleviated to only a limited degree, the administration of four *P*. *acidilactici* strains effectively regulated the gut flora and provided a potential health benefit to the host, especially the bacteriocin-producing *P*. *acidilactici* strains. Future work should be directed towards the characterization of bacteriocins produced by *P*. *acidilactici* strains, and the exploration of the interaction between bacteriocins and the intestinal flora in mice.

## Data Availability Statement

The raw data supporting the conclusions of this article will be made available by the authors, without undue reservation. The data presented in the study are deposited in the SRA repository, accession number (PRJNA705063).

## Ethics Statement

The animal study was reviewed and approved by The Ethics Committee of Jiangnan University, China (JN. No 20171219–20180129 [169]), and this experiment was conducted according to the European Community guidelines (Directive 2010/63/EU).

## Author Contributions

YQ, ZQ, QZ, and WC were responsible for conceptualization, methodology and software. YQ, ZQ, FT, LY, JZ, and HZ were responsible for data curation, writing-original draft preparation, visualization, and investigation. QZ and WC were responsible for supervision and validation. YQ, ZQ, QZ, and WC were responsible for writing-reviewing and editing. All authors contributed to the article and approved the submitted version.

## Funding

This work was supported by the National Natural Science Foundation of China Program (31820103010, 31871773, and U1903205); the Key Scientific and Technological Research Projects in the Key Areas of the Xinjiang Production and Construction Corps (2018AB010); National First Class Discipline Program of Food Science and Technology (JUFSTR20180102); the BBSRC Newton Fund Joint Centre Award; and Collaborative Innovation Center of Food Safety and Quality Control in Jiangsu Province.

## Conflict of Interest

The authors declare that the research was conducted in the absence of any commercial or financial relationships that could be construed as a potential conflict of interest.
